# miRNome Profiling in Bicuspid Aortic Valve-Associated Aortopathy by Next-Generation Sequencing

**DOI:** 10.3390/ijms18112498

**Published:** 2017-11-22

**Authors:** Andrea Borghini, Ilenia Foffa, Silvia Pulignani, Cecilia Vecoli, Lamia Ait-Ali, Maria Grazia Andreassi

**Affiliations:** CNR Institute of Clinical Physiology, 56124 Pisa, Italy; ilenia@ifc.cnr.it (I.F.); pulignani@ifc.cnr.it (S.P.); vecoli@ifc.cnr.it (C.V.); lamia@ifc.cnr.it (L.A.-A.); andreassi@ifc.cnr.it (M.G.A.)

**Keywords:** miRNome, NGS, BAV, TAV, aortopathy

## Abstract

The molecular mechanisms underlying thoracic aortic aneurysm (TAA) in patients with bicuspid aortic valve (BAV) are incompletely characterized. MicroRNAs (miRNAs) may play a major role in the different pathogenesis of aortopathy. We sought to employ next-generation sequencing to analyze the entire miRNome in TAA tissue from patients with BAV and tricuspid aortic valve (TAV). In the discovery stage, small RNA sequencing was performed using the Illumina MiSeq platform in 13 TAA tissue samples (seven patients with BAV and six with TAV). Gene ontology (GO) and KEGG pathway analysis were used to identify key pathways and biological functions. Validation analysis was performed by qRT-PCR in an independent cohort of 30 patients with BAV (26 males; 59.5 ± 12 years) and 30 patients with TAV (16 males; 68.5 ± 9.5 years). Bioinformatic analysis identified a total of 489 known mature miRNAs and five novel miRNAs. Compared to TAV samples, 12 known miRNAs were found to be differentially expressed in BAV, including two up-regulated and 10 down-regulated (FDR-adjusted *p*-value ≤ 0.05 and fold change ≥  1.5). GO and KEGG pathway enrichment analysis (FDR-adjusted *p*-value < 0.05) identified different target genes and pathways linked to BAV and aneurysm formation, including Hippo signaling pathway, ErbB signaling, TGF-beta signaling and focal adhesion. Validation analysis of selected miRNAs confirmed the significant down-regulation of miR-424-3p (*p* = 0.01) and miR-3688-3p (*p* = 0.03) in BAV patients as compared to TAV patients. Our study provided the first in-depth screening of the whole miRNome in TAA specimens and identified specific dysregulated miRNAs in BAV patients.

## 1. Introduction

Dilatation of the aortic root and ascending aorta is frequently present in patients with bicuspid aortic valve (BAV) and can lead to complications, including aneurysm formation and aortic dissection [1].

Aortic diameter is currently the major clinical risk criterion used in combination with other potential risk factors (e.g., family history of dissection, presence of coarctation, root phenotype) to guide elective surgical repair of ascending aortic aneurysms in BAV patients [2].

However, the size criterion is an imperfect predictor of aortic dissection or rupture in many patients who suffer aortic complications at smaller diameters [3].

As a consequence, additional predictors (e.g., imaging, genetic or biological markers) are needed to identify individualized therapeutic and surgical strategies [4]. Previous studies of global gene expression and proteomic profiles have shown differences between the molecular processes of aorta dilatation in BAV and tricuspid aortic valve (TAV) patients, but the mechanisms underlying the aortopathy remain uncharacterized [5,6].

MicroRNAs (miRNAs) are short non-coding RNAs (~22 nucleotides) that negatively modulate gene expression at the post-transcriptional level through binding to the 3′UTR of their target mRNA [7]. Based on their long half-life in blood and selective expression in certain tissues, miRNAs can be key modulators in the development of aortic aneurysm as well as potential circulating biomarkers in the progression of BAV aortopathy [4,8].

Several studies have examined miRNA expression levels in aortic tissue specimens collected from patients with ascending thoracic aortic aneurysm (TAA) associated with BAV or TAV, using microarray or quantitative real-time PCR methods [9,10,11,12,13,14,15,16,17].

In recent years, the advent of next-generation sequencing (NGS) provides the opportunity to examine the entire miRNome signature and to identify miRNAs expressed at levels below the microarray’s detectable threshold as well as discovering novel miRNAs [18].

To date, comprehensive profiling of miRNAs through deep sequencing in patients with BAV has not yet been attempted. Therefore, the purpose of this study was to perform miRNA profiling in TAA specimens from BAV and TAV patients using NGS.

## 2. Results

### 2.1. miRNA Profiling by Next-Generation Sequencing

**Figure 1:**
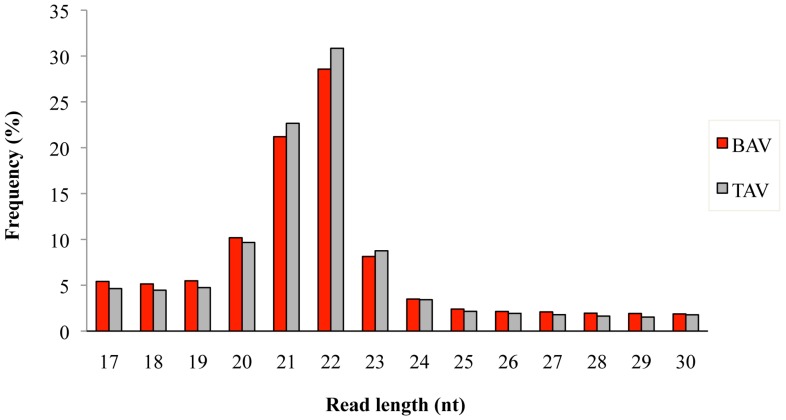
Length distribution of mapped sequence reads in bicuspid aortic valve (BAV) and tricuspid aortic valve (TAV) samples.

A total of 3.3 × 10^6^ and 2.6 × 10^6^ reads were obtained from BAV (*n* = 7) and TAV (*n* = 6) patients, respectively. After removing the adaptor sequences, low-quality tags, contaminants, and reads of <17 nt and >30 nt, we obtained an average sequence read of 97.9% for BAV and 97.6% for TAV, respectively. The length distribution of mapped reads peaked from 20 to 23 nucleotides in both groups of patients (Figure 1), which represents the length of the majority of miRNAs. The most abundant size class was 22 nt, which accounted for 28.5% and 30.8% of the total reads in BAV and TAV samples, followed by 21 nt (21.1% in BAV and 22.6% in TAV). A total of 489 known human mature miRNAs was identified by using iMir software and 12 of them (2.4%) were found to be significantly differentially expressed. Of these, two miRNAs were up-regulated and ten down-regulated in the BAV group, as shown in Table 1.

Bioinformatic analysis identified five novel miRNAs, as predicted from their hairpin secondary structure features derived from human genomic sequences. They were most likely orthologs of miRNAs found in other systems, displaying 100% seed identity (Table 2). However, they did not show significant differential expressions between the BAV and TAV groups.

### 2.2. Functional Analysis

KEGG pathway enrichment analysis revealed that the set of differentially expressed miRNAs are associated with 45 different pathways (FDR-adjusted *p*-value ≤ 0.05), among which 9 pathways (Hippo signaling pathway, ErbB signaling, ubiquitin mediated proteolysis, TGF-beta signaling, Wnt signaling, tight junction, regulation of actin cytoskeleton, focal adhesion, and PI3K-Akt signaling pathways) were known to be associated with the pathogenesis of BAV and/or aortopathy.

Hippo signaling pathway (*p* = 1.1 × 10^−5^), ErbB signaling (*p* = 6.4 × 10^−5^), ubiquitin mediated proteolysis (*p* = 0.001), TGF-beta signaling (*p* = 0.006) and Wnt signaling (*p* = 0.009) were the most significantly enriched pathways.

**Figure 2:**
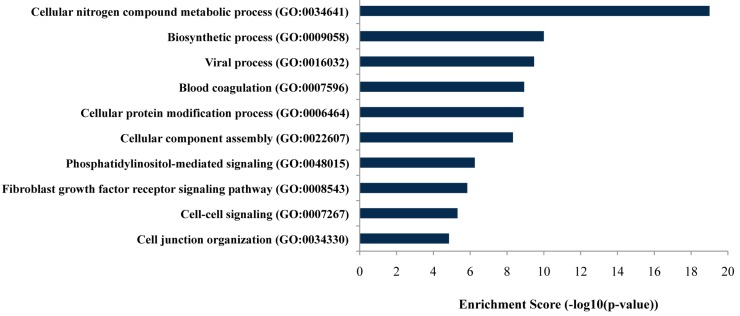
Bar plot ranking of the top ten biological processes based on enrichment score by gene ontology (GO) analysis.

As listed in Figure 2, gene ontology (GO) analysis revealed that targeted genes of the differentially expressed miRNAs were mainly enriched in genes related to specific biological processes, including the cellular nitrogen compound metabolic process and biosynthetic process (FDR-adjusted *p*-values ≤ 0.05).

### 2.3. Validation of Selected microRNAs Using qRT-PCR

**Figure 3:**
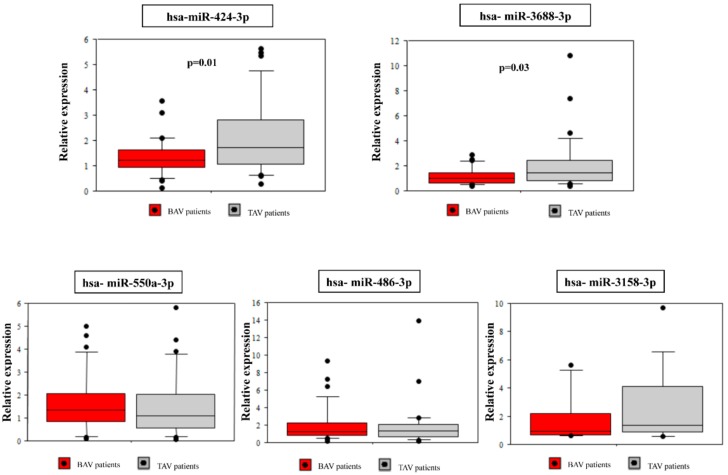
qRT-PCR validation of selected differentially expressed miRNAs in BAV and TAV samples.

Among the 12 differentially expressed miRNA between the BAV and the TAV group, five highly significant dysregulated (miR-424-3p, miR-486-3p, miR-550a-3p, miR-3158-3p, miR-3688-3p) whose target genes were significantly enriched in functional pathways related to BAV aortopathy were selected for further validation. Demographic and clinical characteristics of the validation cohort are reported in Table 1. As expected, there were significant differences between BAV and TAV patients regarding age (*p* = 0.003), gender (*p* = 0.003) and aortic diameter (*p* = 0.002). The BAV group included 10 (33%) patients with aortic valve insufficiency, 13 (43%) patients with stenosis and seven (23%) patients with both aortic stenosis and valve insufficiency. Nearly all TAV patients (83%) had aortic valve insufficiency. qRT-PCR confirmed the significant down-regulation of miR-424-3p (*p* = 0.01) and miR-3688-3p (*p* = 0.03) in BAV patients as compared to TAV patients. The miR-3158-3p showed a decreased expression in BAV patients consistent with the sequencing results, but without statistical significance (Figure 3). The other two (miR-486-3p and miR-550a-3p) did not show significant differences in the expression between the two groups (Figure 3).

There was no statistically significant correlation between the aortic diameter and the expression of any miRNAs in both BAV and TAV group (*p* ≥ 0.05).

In the BAV group, the differentially expressed miRNAs were not significantly different in patients with aortic stenosis as compared to aortic insufficiency (*p* ≥ 0.05). Additionally, no significant differences were observed in severely dilated tissues compared to less dilated samples when miRNA expression findings were stratified based on aortic diameter (≥50 mm) of the TAAs at the time of surgical resection.

## 3. Discussion

To our knowledge, this is the first study to characterize the entire miRNome signature for distinguishing TAA specimens from BAV and TAV patients.

Collectively, we identified 12 miRNAs significantly differentially expressed in the BAV group. Functional analysis revealed that these miRNAs were significantly involved in the regulation of genes related to pathways that govern valve development and function. After selection of highly dysregulated miRNAs, the validation analysis showed that both miR-424-3p and miR-3688-3p were significantly down-regulated in BAV patients, according to the sequencing results. A similar pattern of down-regulation was observed for miR-3158-3p, while the other two miRNAs (miR-486-3p, miR-550a-3p) showed no differences.

Previous studies have compared differential expression of miRNAs in TAA obtained from patients with BAV or TAV [10,11,12,13,14,15,16,17]. Generally, the vast majority of miRNA-related research was conducted on aneurysmal aortic tissue as compared to unaffected aortic specimens, excluding patients with BAV [9,10,11,12,16].

A combined list of observed differences between thoracic aneurysm and control aortic tissues contained a total of 208 miRNAs [9,10,11,12,14,16]; however, only 10 (5%) were seen in more than one study and the results agreed in the direction of change (up or down). For 20 (10%) microRNAs the results disagreed in the direction of change, and the majority (178; 85%) of the microRNAs were seen in only one study.

Several factors such as study design, subtypes and number of samples, as well as differences in miRNA profiling platforms may complicate the comparison of results obtained from miRNA studies in TAA.

Only one study examined the expression of a set of miRNAs (miRA-1, miR-21, miR-29a, miR-133a, miR-143, miR-145) in both TAA tissue and plasma obtained from patients with BAV and TAV at the time of surgical resection [15]. Significant difference was found in miR-1 and miR-21 abundance between BAV and TAV only in aortic tissue samples [15].

Recently, a microarray screening investigated differential miRNA expression in same-patient paired samples of aortic tissue from severely dilated and less dilated aortic segments from BAV patients [13]. Twenty-one miRNAs showed a trend for different expression between less and severely dilated segments. Specifically, the authors validated a significantly increased expression of the miR-17 gene cluster members (miR-18a, miR-19a/b) and other miRNAs that share the miR-17 seed sequence (miR-17, miR-20a/b, miR-106a/b, miR-93) in normal-appearing BAV less dilated tissue or normal aortic samples.

A very recent study identified unique profiles of differentially expressed miRNA in the aortic convexity and concavity of mildly dilated aorta of BAV and TAV patients compared to donor aortic samples [17], with a maximum of two common miRNAs between BAV and TAV in the aortic convexity (up-regulated miR-99a-5p and miR-199a-5p in both TAV and BAV aortic concavity).

Finally, a global miRNA expression profiling identified that miR-122, miR-130a and miR-486 differed significantly between TAV and BAV patients [19]. An increased ascending aorta diameter was associated with the down-regulation of plasma expression of miR-718, supporting it as a biomarker of aortic dilation [19]. In our NGS study, only two differentially expressed miRNAs (miRNA 203a-3p, miRNA 377-5p) have been previously associated with either TAA or BAV aortopathy in the same direction [10,11].

However, it is also interesting to note that several “sister” miRNAs differentially expressed in our work (miRNA486-3p, miRNA 210-5p, miRNA-340-3p, miRNA 424-3p) have been previously found deregulated in other studies [9,10,11,14,17,19].

Importantly, recent reports have indicated that the “sister” miRNA species (so-called because they generate from the same miRNA precursor) can be functional and implicated in the pathology of several types of disease through co-regulating a common set of genes [20].

In particular, miRNA-424 (or miR-424-5p), which shares the same miRNA precursor with the validated miR-424-3p in our study, plays an important role in post-ischemic vascular remodeling and angiogenesis [21]. Additionally, a recent study has shown that levels of miR-424 were lower in the patients with aortic stenosis than in the control, and miR-424 was statistically correlated with several hemodynamic parameters including aortic valve area, mean aortic gradient and peak flow velocity [22].

In the report by Liao et al. (2011), miR-424 was also found to be down-regulated in aneurysmal tissues compared with normal wall tissues [10].

Finally, and most importantly, miR-424 has been shown to target the activity of the TGF-β signaling pathway [23,24], supporting the notion that the epigenetic alterations associated with TGF-signaling may be a key player in the pathogenesis of thoracic aortic dilatative disease. Altogether, these studies strongly indicate a key role for miRNAs in the pathogenesis of ascending aorta dilatation. However, at present, no miRNA can be useful as a biomarker in clinical practice. A rigorous comparison between studies is still needed to translate basic research into the clinic arena.

We acknowledge that our study has several limitations. Firstly, we performed technical validation of only five selected miRNAs and there was an overall poor correlation between findings using both qRT-PCR and NGS. The validation rate for our NGS data by qRT-PCR was 40%, comparable to the rate of 30–40% reported for miRNA hybridization microarray [25]. Indeed, it is well-known that RT-PCR is not an infallible validation method for measuring miRNAs as previously reported by others [26,27]. Secondly, the composition of the validation cohort was less balanced in terms of difference in risk factors when compared to the initial cohort. We cannot exclude that the detection of significant differences in miRNA expression profiles can be limited by these confounding factors. Thirdly, it was not possible to analyze the link between the differential expression of miRNAs and target genes. Biological studies with gain/loss-of-function and mimics/inhibitor should also be performed to completely validate their functions. Despite these limitations, this study describes advances of particular importance, since for the first time we applied the NGS approach which offers several advantages over both qRT-PCR and microarray assay, such as high sensitivity towards low abundant transcripts, excellent reproducibility and the possibility of discovering unknown miRNAs.

## 4. Materials and Methods

### 4.1. Study Population and Tissue Samples

All patients had undergone elective surgical repair for TAA, with or without concurrent aortic valve replacement (AVR). In all patients, the morphology and function of the aortic valve was assessed by preoperative echocardiography. Aortic valve stenosis and aortic insufficiency of a moderate or severe degree was evaluated by using the established American Society of Echocardiography criteria [28]. Ascending aortic diameters were assessed by computed tomography or magnetic resonance imaging. Valve morphology (TAV/BAV) was confirmed by intra-operatory observation.

A total of 13 consecutive patients (7 patients with BAV and 6 with TAV) were included in the discovery phase. Concomitant AVR was required in 11 patients (7 BAV patients and 4 TAV patients). In the validation phase, candidate miRNAs were tested by quantitative real-time PCR (qRT-PCR) in an independent cohort of 30 patients with BAV (26 males; 59.5 ± 12 years) and 30 patients with TAV (16 males; 68.5 ± 9.5 years). Concomitant AVR was required in 44 patients (26 BAV and 18 TAV patients).

Samples of the aneurysmal wall of the thoracic aorta were obtained during the surgical procedure. After aneurysm excision, ascending aortic tissue was immediately stored in liquid nitrogen until analysis. A complete medical history, including traditional cardiovascular risk factors was collected from all patients (Table 3). Aortic samples from patients with aneurysms secondary to genetic syndromes such as Marfan, Ehlers-Danlos, and Loeys-Dietz or with other cardiovascular diseases, cancer or chronic diseases were excluded from this study. The study was approved by the Massa Ethical Committee on 19 September 2013 (Study Protocol No. 436). All subjects gave written informed consent in accordance with the Declaration of Helsinki.

### 4.2. RNA Extraction and Quantification

Total RNA was extracted from TAA tissue specimens using the miRNeasy Mini Kit (Qiagen, Milan, Italy) according to the manufacturer’s recommendations. The RNA concentration of each sample was determined with a Nanodrop Lite spectrophotometer (Thermo Scientific, Waltham, MA, USA), and the quality of RNA was assessed using the RNA 6000 Nano Kit with Bioanalyzer 2100 (Agilent Technologies, Santa Clara, CA, USA). Only samples with RNA Integrity Number (RIN) ≥ 7.0 were subsequently analyzed.

### 4.3. miRNA Sequencing and Data Analysis

For sequencing, 1 μg of total RNA was used to generate the small RNA sequencing library, according to the NEBNext^®^ Multiplex Small RNA Library Prep Set for Illumina protocol (New England BioLabs, Hitchin, UK). Briefly, a pair of adaptors was ligated sequentially to the 3′ and 5′ ends of miRNA, and following ligation with adaptors, a library was prepared by reverse transcription and PCR preamplification. The quality of the library was measured by the Agilent Bioanalyzer 2100 (Agilent Technologies, Santa Clara, CA, USA) using High-Sensitivity DNA chips. cDNA with concentrations of higher than 1 nM and no dimmer contamination was loaded into the Illumina MiSeq sequencer (Illumina Inc., San Diego, CA, USA), using v2 chemistry (50 cycles). The final reads of miRNAs were identified by normalization with the total reads of all called miRNAs in the sample. Sequencing data was trimmed to remove adapter sequences and aligned against a human reference database (miRBase v20) using iMir software, a reliable, flexible and fully automated workflow, useful for rapidly and efficiently analyzing high-throughput small RNA-Seq data [29].

Identification and analysis of differentially expressed miRNAs was implemented in iMir, based on quantile normalization and Fisher’s exact test. A miRNA was considered differentially expressed when showing an absolute fold change (FC) of 1.5 or greater between BAV and TAV patients, with an FDR-adjusted *p*-value ≤ 0.05.

The prediction of novel human microRNAs was performed in iMir with miRanalyzer and miRDeep 2 stand-alone tools. In order to predict the orthology relationships among organisms, the novel identified mature miRNAs were aligned to mature miRNA sequences deposited in miRBase v20 by using the SSEARCH alignment algorithm from the FASTA package. Results were filtered for the expectation value (*e*-value) <  0.05 and nucleotide overlap  ≥15.

### 4.4. Target Prediction and Enrichment Pathway Analysis

In order to understand the potential biological functions of differentially expressed miRNAs, their putative targets and pathways were predicted by using DIANA-miRPath software (http://microrna.gr/mirpath). DIANA-miRPath v3.0 extends the Fisher’s Exact Test, EASE score and False Discovery Rate (FDR) methodologies, with the use of unbiased empirical distributions [30]. Gene ontology (GO) analysis of the potential target genes was based on the terms of the GO database: gene function-related biological processes were detected, and genes with similar functions were combined. GO and pathway enrichment analyses with an adjusted *p*-value or FDR ≤ 0.05 were considered to be significantly enriched.

### 4.5. Quantification by qRT-PCR

qRT-PCR validation using selected miRNA, chosen on fold-change or statistically significant NGS findings and the results of pathway enrichment analysis, was performed an independent cohort of 60 aortic tissues collected from 30 BAV to 30 TAV patients. cDNA synthesis was performed by using TaqMan miRNA Reverse Transcription Kit using TaqMan Universal PCR Master Mix, no AmpErase UNG (Thermo Fisher Scientific). qRT-PCR reactions were run in triplicate for each sample using a 384-well plate CFX RT-PCR System (Bio-Rad, Milan, Italy). The expression levels of miRNAs were normalized to U6 snRNA (ID:001973) by using the ΔΔ*C*t method.

### 4.6. Statistical Analysis

Statistical analyses of the data were conducted with the StatView statistical package, version 5.0.1 (Abacus Concepts, Berkeley, CA, USA). Values are presented as mean ± standard deviation (SD), median (interquartile range) or percent. Differences in non-continuous variables were tested by χ^2^ analysis. Differences between the means of the two continuous variables were evaluated by the Student’s *t*-test. Any differential expression of miRNAs between groups was determined by the Mann-Whitney *U*-test. Regression analysis was used to characterize relationships between the expression of miRNAs and diameter score. Values of *p* < 0.05 were considered statistically significant.

## 5. Conclusions

In summary, the current study has described the entire miRNome of BAV and TAV patients. Bioinformatic analysis indicated that miRNA target genes and pathways may play a key role in the pathogenesis of TAA. However, due to the limited known function of these miRNAs, further work must be carried out to gain more knowledge of the mechanisms of disease associated with their dysregulation as well as defining the clinical perspectives of miRNA research in aortopathy.

## Figures and Tables

**Table 1:** List of microRNAs (miRNAs) significantly up-regulated and down-regulated in the BAV group.

miRNAs	FDR-Adjusted *p*-Value	Fold Change
miR-203a-3p	0.031	1.83
miR-377-5p	0.030	2.15
miR-5684	0.036	−4.64
miR-664b-3p	0.021	−2.35
miR-424-3p	0.006	−2.55
miR-550a-3p	0.011	−3.24
miR-3158-3p	0.009	−4.35
miR-3688-3p	0.017	−3.83
miR-511-3p	0.046	−2.36
miR-340-3p	0.033	−2.11
miR-210-5p	0.018	−2.98
miR-486-3p	0.016	−5.04

**Table 2:** Summary of putative novel miRNAs detected by small RNA sequencing.

miRNA Candidate	Sequence (5′-3′)	Chromosome Position	ΔG (kcal/mol)	*Orthologous* miRNA
1	TAAGGGCTGGGTCGGTTGGGCTGGG	2:133038057-133038161	−40.30	*mmu-miR-6937-5p*, *bta-miR-2487*
2	GGAGCCTCGGTTGGCCTCGGATAGC	22:22210613-22210687	−22.50	*bta-miR-2904*
3	GTGGAGGACTGAGAAGGTGAGGC	5:76376302-76376432	−43.00	*mmu-miR-1940*
4	AAAGGTAGATAGAACAGGTCTTGTT	15:83424755-83424829	−21.60	*bta-miR-1839*, *cgr-miR-1839-5p*, *cfa-miR-1839*, *mmu-miR-1839-5p*, *eca-miR-1839*, *ssc-miR-1839-5p*, *rno-miR-1839-5p*, *chi-miR-1839*
5	GCCGGCGGGAGCCCCGGGGAGAGT	5:71146764-71146846	−33.90	*mmu-miR-2137*

**Table 3:** Clinical and demographic characteristics of study population.

Variables	Discovery Cohort			Validation Cohort		
	BAV (*n* = 7)	TAV (*n* = 6)	*p* Value	BAV (*n* = 30)	TAV (*n* = 30)	*p* Value
Age (years ± SD)	56.2 ± 17	61.6 ± 10	0.5	59.5 ± 12	68.5 ± 9.5	0.003
Gender, male (*n*, %)	7 (100%)	4 (66%)	0.09	26 (86%)	16 (53%)	0.003
Ascending aortic diameter (mm)	48.8 ± 3.2	48.5 ± 3.8	0.8	49.5 ± 5	53.7 ± 4.9	0.002
Aortic Stenosis (*n*, %)	6 (86%)	1 (16%)	0.01	13 (43%)	0 (0%)	<0.0001
Aortic valve insufficiency (*n*, %)	1 (14%)	5 (83%)	0.01	10 (33%)	25 (83%)	<0.0001
Hypertension (*n*, %)	4 (57%)	3 (50%)	0.8	18 (60%)	21 (70%)	0.3
Smoking habit (*n*, %)	4 (57%)	1 (16%)	0.1	7 (23%)	3 (10%)	0.2
Diabetes (*n*, %)	1 (14%)	0 (0%)	0.3	0 (0%)	2 (6%)	0.1
Dyslipidemia (*n*, %)	0 (0%)	1 (16%)	0.5	7 (23%)	9 (30%)	0.5
